# Radial glia at the neurovascular interface during cortical development

**DOI:** 10.3389/fncel.2026.1788096

**Published:** 2026-02-20

**Authors:** Njoud Al-Naama, Caroline Alayne Pearson

**Affiliations:** Center for Neurogenetics and the Feil Family Brain and Mind Research Institute, Weill Cornell Medicine, New York, NY, United States

**Keywords:** angiogenesis, cortex development, endothelial cell (EC), neural stem/progenitor cells, neurogenesis

## Abstract

Radial glia are a specialized population of neural progenitor cells that persist throughout embryogenesis and into adulthood. Throughout this period, radial glia reside in a highly dynamic microenvironment that influences various biological decisions that govern typical cortical development. Subsequently, radial glia must fine-tune their responses to numerous environmental cues throughout development. The establishment of the cortical vasculature coincides with neurogenesis and dramatically alters the radial glia microenvironment by increasing oxygen and metabolite delivery. In addition, a synergistic spatial relationship between radial glia and endothelial cells regulates multiple aspects of radial glial biology. Here, we discuss crosstalk between radial glia and the cortical vasculature/endothelial cells throughout development, including the influence of extrinsic angiogenic processes and our growing understanding of the intricate spatial relationships between radial glia and endothelial cells.

## 1 Introduction

**Figure 1:**
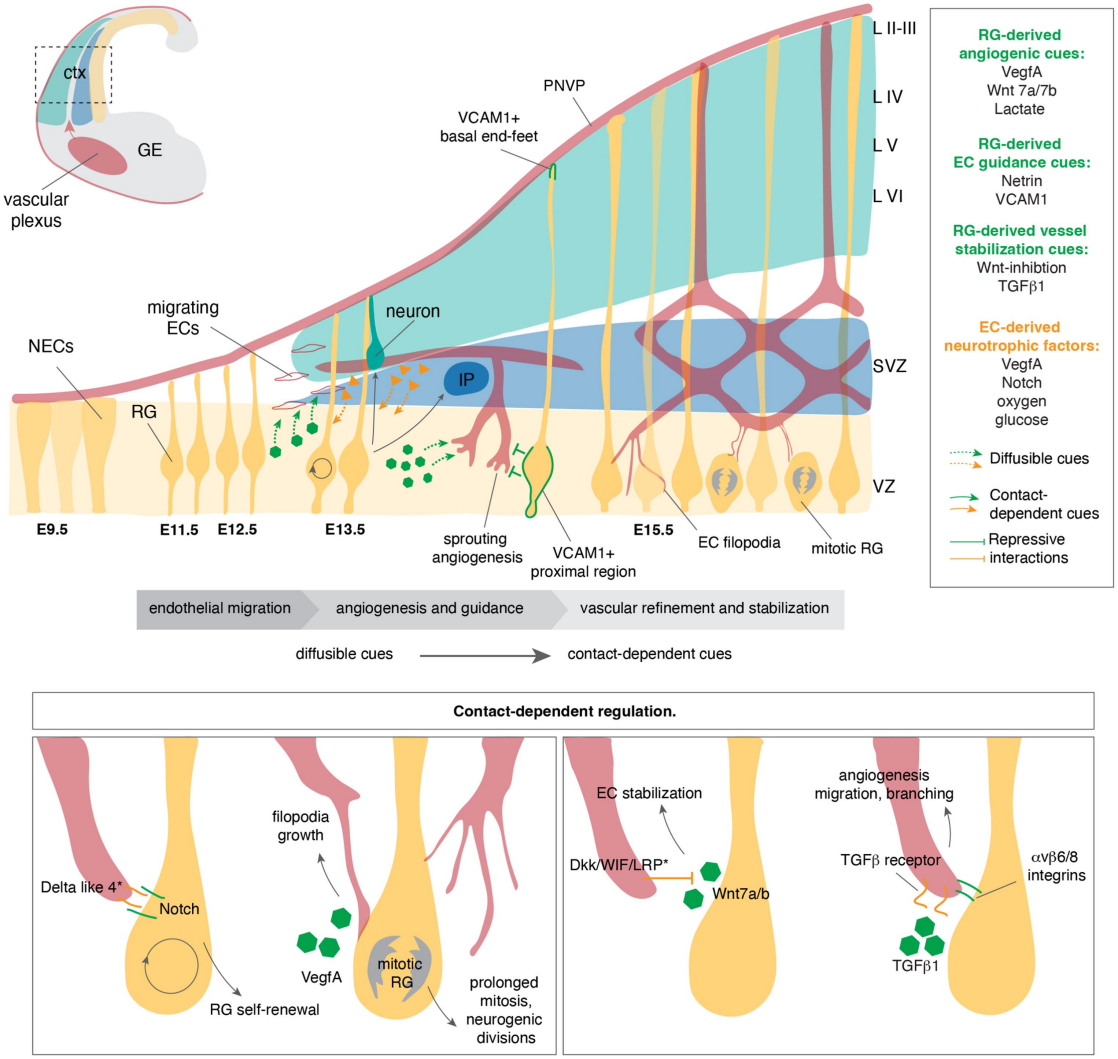
Bidirectional, stage-specific interactions between radial glia and endothelial cells during cortical development. Schematic illustrating how radial glia (RG) and endothelial cells (ECs) coordinate endothelial migration, angiogenesis, vascular stabilization, and neurogenesis through a combination of diffusible and contact-dependent cues across embryonic day (E) 9.5 to E15.5. Early RG provide diffusible angiogenic and migratory cues, including VegfA, Wnt7a/b, and lactate, to promote sprouting angiogenesis and growth. RG provide guidance cues including Netrin and VCAM1, that direct EC migration and positioning. In parallel, ECs supply neurotrophic and metabolic factors to RG, including Vegf, Notch ligands, oxygen, and glucose, supporting RG self-renewal and differentiation. The perineural vascular plexus (PNVP) is in direct contact with RG via VCAM1+ basal end feet. The bottom panel further details contact-dependent signaling. Direct RG-EC interactions regulate RG behavior and vascular maturation. Delta-like/Notch signaling promotes RG self-renewal, while VegfA supports EC filopodia expansion near mitotic RG. Wnt inhibition contributes to EC stabilization, while TGFB1 signaling is localized to the RG-EC interface via integrins to modulate angiogenesis, migration, and vessel stabilization. Asterisks denote potential candidates that may mediate the interaction. Distinct modes of signaling are indicated: dashed arrows denote diffusible cues, solid arrows denote contact-dependent cues, and blunt-ended lines represent repressive signaling. Orange triangles represent EC-derived cues, green hexagons represent RG-derived cues. ctx, cortex; GE, ganglionic eminence; PNVP, perineural vascular plexus; ECs, endothelial cells; NECs, neuroepithelial cells; RG, radial glia; IP, intermediate progenitors; VZ, ventricular zone; SVZ, subventricular zone; LII-VI, cortical layers II-VI.

The formation of the cerebral cortex requires exquisitely coordinated interactions between neural progenitor cell (NPC) populations and their surrounding microenvironment. As the embryonic brain expands, diverse extrinsic cues, including metabolites and oxygen, and emerging vasculature, intersect with intrinsic lineage programs to shape the timing and trajectory of cortical development (Figure 1). Among these progenitors, radial glia (RG) serve as the primary NPCs of the developing cortex, generating nearly all excitatory neurons and glia (Taverna et al., 2014; Beattie and Hippenmeyer, 2017).

While we now know that RG are NPCs, not glia, they do share characteristics with glial cells, which has led to the persistence of the term. RG have long processes that span the developing brain and provide scaffold-like support for migrating neurons. Furthermore, they express “glial” markers, including GFAP, BLBP, and GLAST, and give rise to astrocytes and oligodendrocytes (Rakic, 2003). Like astrocytes, RG are intimately associated with the vasculature, although crosstalk between RG and endothelial cells (ECs) remains less well characterized. This is in part due to technical difficulties in distinguishing between lineage-specific, cell-autonomous, and non-cell-autonomous roles.

During brain development, neurogenesis and angiogenesis are temporally and spatially coordinated, and RG-EC interactions are critical to our understanding of how the brain develops. Emerging evidence also offers insights into the importance of RG-EC communication in the maintenance and function of the cortical vasculature and NPC niche. Disruption of RG–EC crosstalk has important implications for neurodevelopment. Perturbations in angiogenic and vascular stabilization pathways result in abnormal cortical vascularization and barrier formation, underscoring the need for coordinated neurovascular maturation. Emerging human stem cell–derived organoid models further implicate altered neurovascular interactions in developmental vascular pathology.

### 1.1 Radial glia balance self-renewal and differentiation to build the adult brain

The early neocortex is initially composed of neuroepithelial cells (NECs), a rapidly dividing epithelial sheet that expands the progenitor pool and contributes to the tangential expansion of the nascent neuroepithelium (Taverna et al., 2014; Chenn et al., 1998). As corticogenesis progresses, NECs transition into RG through transcriptional and morphological changes that establish neurogenic competence while maintaining apical-basal polarity (Figure 1) (Rakic, 2003; Paridaen and Huttner, 2014). In higher mammals, including humans, there are different subtypes of RG, distinguished primarily by their morphology and spatial location (Molnár et al., 2019; Lui et al., 2011; Micali et al., 2023; Nowakowski et al., 2016; Dehay and Huttner, 2024). In this review, we will focus on RG in the ventricular zone (VZ), termed apical radial glia (aRG) or ventral radial glia (vRG) in the human cortex (Lui et al., 2011). Here, we will refer to this population as RG regardless of species.

In the mouse, the transition to RG occurs around embryonic day (E) 11.5 and establishes the primary NPC population of the developing cortex (Figure 1). Following their emergence, RG initially divide symmetrically to amplify the progenitor pool. From E13.5 onwards, RG begin to execute asymmetric divisions to generate an intermediate progenitor (IP) or a neuron. IPs reside in the subventricular zone (SVZ), while nascent neurons migrate along the basal processes of the daughter RG into the cortical plate (Figure 1). From E16.5, RG generate astrocytes and subsequently oligodendrocyte progenitor cells (Taverna et al., 2014; Beattie and Hippenmeyer, 2017; Molnár et al., 2019; Lui et al., 2011). Postnatally, a subset of RG transition into type B stem cells of the VZ/SVZ of the lateral ventricle, which persist into adulthood (Obernier and Alvarez-Buylla, 2019). Thus, RG maintain “stemness” to ensure a population persists into adulthood while tightly regulating neurogenic and gliogenic transitions to generate all three neural lineages that comprise the adult cerebral cortex. Throughout development and into adulthood, NPCs maintain close, physical interactions with the vasculature (Doetsch, 2003).

### 1.2 Radial glia are temporally attuned to environmental signals

RG do not maintain a static molecular identity throughout development; they transition through successive transcriptional states (Telley et al., 2019; Dong et al., 2022). Temporal single-cell analyses show that early RG (E11.5–E12.5) occupy intrinsically driven cell-cycle dominated states characterized by proliferative and chromatin-regulatory signatures. As development progresses, RG transition toward transcriptional programs enriched for receptors, adhesion molecules, extracellular matrix interactors, and components of major developmental signaling pathways (E13.5 onwards) (Telley et al., 2019; Dong et al., 2022; Eze et al., 2021). This shift reflects an expansion in the molecular machinery required for environmental sensing, temporally aligned with the establishment of the cortical vasculature. While RG appear transcriptionally primed to sense microenvironmental cues, it remains unclear whether they consistently respond to these signals or, in some contexts, selectively ignore them.

### 1.3 Establishment and maturation of the cortical vasculature

From E8.5 to E10, a blood vessel network termed the perineural vascular plexus (PNVP) envelops the entire neural tube (Paredes et al., 2018). At E11.5, ECs from a plexus adjacent to the lateral ganglionic eminence migrate into the dorsal telencephalon (Figure 1). Subsequently, the cortical vasculature undergoes a significant elaboration to perfuse the growing neocortex (Figure 1) (Vasudevan et al., 2008; Rosenstein et al., 2010; Tata and Ruhrberg, 2018). Spatial transcriptomic data localize vascular cells to the VZ and SVZ, where they lie in immediate proximity to RG, basal RG, and IPs, reinforcing the theory that angiogenesis and progenitor dynamics unfold within a shared, rapidly shifting microenvironment (Wang et al., 2025). The emergence and expansion of vascular structures introduce local variation in oxygen tension, metabolite availability, and paracrine signals, altering the extracellular landscape that RG encounters (Figure 1). We previously reviewed the literature associated with the influence of metabolite availability and oxygen tension on RG development (Andrews and Pearson, 2024); therefore, in this review, we will focus on the direct interactions between RG and ECs and their co-development.

## 2 Molecular mechanisms of neural-endothelial crosstalk

Bidirectional interactions between NPCs and ECs coordinate cortical vascularization, with RG playing a central organizing role. RG shape cortical vascularization through multiple, temporally coordinated roles that extend beyond the initial establishment of a growth-permissive substrate. First, RG establish an angiogenic environment that enables endothelial entry into the developing cortical wall. Second, RG impose spatial and positional instructions that guide endothelial growth toward progenitor-rich niches. Third, RG actively refine and stabilize nascent vascular networks to ensure their persistence and functional integration with the expanding cortex (Figure 1).

### 2.1 Radial glia provide early pro-angiogenic cues

Before endothelial sprouts invade the cortical wall, RG establish a permissive angiogenic microenvironment that enables vascular entry and orients growth toward progenitor-rich zones. A major regulator of this process is Vascular Endothelial Growth Factor A (VegfA). Genetic reduction of *Vegfa* in the murine neural tube disrupts forebrain vascular ingression and directional growth into the developing forebrain, indicating that progenitor-derived VegfA is functionally required for early cortical vascularization (Haigh et al., 2003; Raab et al., 2004). Thus, establishing RG as an instructive source of angiogenic cues rather than passive structural elements awaiting perfusion.

Within this reciprocal framework, Vegf-Vegf receptor (VegfR) signaling functions as a spatial gatekeeper of RG-EC interactions by organizing spatial and metabolic properties of the vascular niche. During early embryonic CNS development (E8.5–E10.5), NPCs express high levels of VegfA, which is sensed by invading ECs through VegfR2. Endothelial responsiveness to this signal is locally constrained by VegfR1, including its soluble decoy isoform, thereby restricting vascular invasion into the VZ and enforcing sharply defined neurovascular boundaries (Haigh et al., 2003; Raab et al., 2004; Takahashi et al., 2015). Disruption of this balance leads to vascular instability and cortical malformations accompanied by altered progenitor behavior, indicating that proper endothelial patterning is essential for maintaining RG organization and fate control (Raab et al., 2004; Li et al., 2013). Thus, VegfA acts by positioning and stabilizing nascent vessels, which in turn regulate RG states through perfusion, metabolic support, and contact-mediated signaling.

In the cortex, VegfA expression in RG is dynamically regulated by the VZ’s metabolic environment. Physiological hypoxia within this compartment stabilizes Hypoxia-Inducible Factor-1α (HIF1α), which enhances *Vegfa* transcription and couples angiogenic initiation to developmental demand (Lange et al., 2016; Forsythe et al., 1996). This process is further refined by the Forkhead domain transcription factor, Foxp1. Early RG express Foxp1 (E12.5), which attenuates HIF1a signaling and represses VegfA expression (Pearson et al., 2020; Buth et al., 2024). Endogenous Foxp1 downregulation from E13.5 onwards is required to upregulate VegfA expression and promote the onset of cortical vascularization and asymmetric RG divisions (Pearson et al., 2020; Buth et al., 2024). Through this mechanism, RG align the timing of vascular entry with the transition to neurogenic divisions, enabling endothelial invasion to relieve hypoxia and sustain continued cortical growth (Lange et al., 2016; Buth et al., 2024).

Glycolytic products have also been linked to vascular ingrowth and RG behavior. Early RG (E11.5) rely on anaerobic glycolysis, generating high levels of lactate (Dong et al., 2022; Lange et al., 2016). In turn, lactate promotes vessel ingrowth into the cortex and RG proliferation by regulating mitochondrial length (Dong et al., 2022). Thus, lactate coordinates vascular growth and neurogenesis in the developing cortex (Figure 1). This study highlighted the need to better understand the role of metabolites as signaling molecules and the complex interplay between metabolism, angiogenesis, and neurogenesis.

The Wnt signaling pathway is a critical mediator of RG-EC communication across corticogenesis. From E10.5, NECs provide a source of Wnt7a and Wnt7b that promote vessel ingression into the cortex. Blockade of Wnt/β-catenin signaling inhibits vessel formation, demonstrating the role of Wnt7a and 7b signaling as potent migration signals in the early cortex (Figure 1) (Daneman et al., 2009; Stenman et al., 2008). Wnt7a also appears to mediate the expression of BBB-specific transporters, including Slc2a1 (Glut1), the primary glucose transporter in ECs. Thus, disrupted Wnt signaling may affect glucose transport into the parenchyma, potentially influencing early cortical development (Andrews and Pearson, 2024; Daneman et al., 2009; Stenman et al., 2008). The use of Wnt signaling in angiogenesis appears to be largely CNS-specific during development, likely reflecting, in part, its role in establishing BBB properties, a defining feature of the CNS.

### 2.2 Radial glia provide positional guidance cues and substrates

Beyond Vegf-driven angiogenic initiation during early corticogenesis, RG provide spatial and positional information that refines endothelial trajectories as vessels penetrate the developing cortical wall (Figure 1). This spatial control is mediated by molecular guidance systems shared by the neural and vascular systems, which constrain where and how ECs sprout and extend within the developing cortical wall (Figure 1) (Carmeliet and Tessier-Lavigne, 2005; Adams and Eichmann, 2010). Among these cues, netrin signaling within the VZ promotes endothelial sprouting through Neogenin/Deleted in Colorectal Carcinoma (DCC)-dependent mechanisms, in which Neogenin and DCC function as netrin receptors, potentially linking classical axon guidance pathways to vascular morphogenesis and angiogenic patterning (Nguyen and Cai, 2006). In the developing brain, netrin expression becomes enriched within the VZ from mid-neurogenesis, coinciding with active progenitor expansion and intraparenchymal vascular growth (Yamagishi et al., 2020). Although multiple neural cell types produce netrins, their enrichment within progenitor-rich regions positions them as candidate mediators of spatial coordination between neurogenesis and angiogenesis (see Table 1).

**Table 1:** Summary of established radial glial-endothelial cell-signaling interactions during cortical development.

Radial Glia → Endothelial cells			
Signal	Stage	Role	References
1. Endothelial migration/entry into the cortex			
VegfA	E8.5-E10.5, E13.5+	Ingrowth into the cortex; angiogenesis.	Haigh et al. (2003), Raab et al. (2004), Takahashi et al. (2015), Lange et al. (2016), Forsythe et al. (1996), Pearson et al. (2020), and Buth et al. (2024)
Lactate	E11.5	Ingrowth into the cortex.	Dong et al. (2022)
Wnt 7a/7b	E10.5	Ingrowth into the cortex, BBB stability.	Daneman et al. (2009) and Stenman et al. (2008)
2. Guidance/adhesion cues			
Netrin	E14.5+	Sprouting angiogenesis	Yamagishi et al. (2020)
VCAM1	E14.5+	Positional cue	Zhang et al. (2020)
3. Vessel stabilization			
Wnt inhibition	E15.5	Vessel stabilization	Ma et al. (2013)
VegfA	E13.5+	Vessel complexity, network elaboration	Lange et al. (2016) and Buth et al. (2024)
TGF-b1	E14.5+	Vessel complexity, network elaboration	Siqueira et al. (2018)

Endothelial cell → Radial Glia			
Signal	Stage	Role	References
VegfA	Primary embryonic NPCs*	Proliferation	Zhang et al. (2003), Jin et al. (2002), and Shen et al. (2004)
Notch	Primary embryonic NPCs*	Proliferation	Shen et al. (2004)

*In vitro.

Categorization of the major bidirectional interactions between RG and EC during cortical development based on direction and function.

RG also shapes cortical angiogenesis through adhesion-dependent mechanisms at the VZ. By generating a polarized adhesive interface, RG impose spatial constraints on where ECs can enter the developing cortical wall. Among known adhesion systems, Vascular cell adhesion molecule 1 (VCAM1) plays a defined role. Its enrichment on RG end-feet forms a molecular surface that restricts endothelial entry to the VZ and preserves the spatial organization of the progenitor niche (Figure 1) (Zhang et al., 2020). Through this basal positioning, VCAM1 provides a contact-dependent layer of positional information, complementing Vegf- and Wnt-mediated paracrine pathways and adding spatial precision to the mechanisms that coordinate vascular ingression during cortical development.

The co-option of axonal guidance pathways to direct endothelial migration is an ongoing field of research. Semaphorins are well-established regulators of angiogenesis and axon guidance, and RG express Sema3a and Sema6a, suggesting their involvement in RG-EC crosstalk (Adams and Eichmann, 2010). However, these studies have focused on neuronal migration. The role of Slit/Robo signaling has been well characterized in cortical development (Yeh et al., 2014; Borrell et al., 2012) and cortical angiogenesis (Jones et al., 2008). Histological analyses have mapped the expression of Slit sources and Robo-expressing neural populations (Marillat et al., 2002), however, the difficulties in dissecting lineage-specific roles *in vivo* has hindered our understanding of this process. Emerging *in vitro* technologies that model neurovascular development (Sun et al., 2023; Sun et al., 2022; Dao et al., 2024; Kistemaker et al., 2025) offer the opportunity to explore the roles of Slit/Robo signaling and other guidance pathways in a lineage-specific manner.

While RG-derived Vegf and guidance cues are sufficient to permit and spatially pattern endothelial invasion of the cortical wall, vascular ingression alone is not synonymous with successful network assembly. As endothelial sprouts penetrate progenitor-rich zones, the angiogenic program must transition from permissive growth to active stabilization to prevent excessive sprouting and regression. This transition necessitates a distinct regulatory role for RG beyond angiogenic initiation, shifting from permissive induction to the enforcement of vascular stability and architectural refinement.

### 2.3 Radial glia promote vascular stability

Following vascular ingression, the role of RG extends beyond permissive signaling for vascular entry to actively shaping nascent network stability and patterning. While Wnt signaling is necessary for the initial ingression and growth of the cortical vasculature (Daneman et al., 2009; Stenman et al., 2008), its inhibition is required for vessel stabilization (Figure 1). The ablation of RG at mid-neurogenic stages (E15.5) causes vessel regression, i.e., rapid loss of cortical vessels, accompanied by ectopic activation of EC Wnt/β-catenin signaling and upregulation of matrix-degrading enzymes, including MMP-2 and MMP-9 (Ma et al., 2013). Ectopic activation of the Wnt signaling pathway in ECs caused the same phenotype, indicating that during mid-embryogenesis, RG are required to inhibit endothelial Wnt signaling to promote vessel stabilization (Ma et al., 2013). *In vitro* experiments further explored this inhibitory relationship, demonstrating that RG-EC contact was critical for downregulating Wnt activity in ECs. However, the inhibitory mechanism involved is unknown, as neural cells continue to express Wnt7a and 7b at these mid-neurogenic stages (Ma et al., 2013). Contact-dependent inhibition of the Wnt pathway can involve Dkk1, WIF-1, and LRP5/6 (Gupta et al., 2021; Steinhart and Angers, 2018); while these factors are known to play a role in angiogenesis in other systems, their roles in mediating vascular stability in the developing cortex remain unknown.

In parallel with this inhibitory control, RG deploy paracrine cues that refine vascular architecture during remodeling. RG-derived transforming growth factor-β1 (TGF-β1) has been shown to regulate cortical angiogenesis, promoting endothelial migration, controlled branching, and network elaboration (Figure 1) (Siqueira et al., 2018). Perturbation of RG-associated TGF-β1 signaling disrupts cortical vascular patterning, whereas enhancement of this pathway increases vessel complexity, supporting a model in which RG supply morphogen-like signals that tune, rather than simply initiate, vascular growth.

Spatial restriction of TGF-β1 signaling within the neurovascular niche is further mediated by RG-expressed αvβ6 and αvβ8 integrins, which locally regulate the activation and bioavailability of latent TGF-β1 and confine signaling to sites of RG-endothelial contact (Figure 1) (Tata and Ruhrberg, 2018; Proctor et al., 2005; McCarty et al., 2005). This RG-dependent integrin β8-TGF-β1 axis may be regulated upstream by sphingosine-1-phosphate receptor (S1pr) signaling in a brain-region-specific manner. S1pr signaling by ventral telencephalic RG controls integrin β8 expression through a Ric8a-dependent intracellular pathway, thereby modulating TGF-β signaling in adjacent vessels and shaping vascular maturation (Ma et al., 2017).

These findings indicate that RG coordinate vascular stability along two axes: suppression of endothelial Wnt/β-catenin signaling to prevent vessel regression, and spatially restricted TGF-β signaling to fine-tune branching and endothelial migration. Through this dual control, RG actively calibrate vascular remodeling in parallel with neuronal fate decisions. This developmental progression positions RG as dual regulators of cortical angiogenesis, initially providing cues to guide vascular entry and stabilization of nascent vessels, a function later assumed by astrocytes, to mediate the maintenance and modulation of vascular integrity in the postnatal brain (Stenman et al., 2008; Ma et al., 2013; Abbott et al., 2006).

### 2.4 Endothelial cells modulate radial glial biology

While RG provide essential cues that initiate and spatially pattern vascular ingression, ECs are not passive recipients of these signals. Instead, newly invading vessels establish an angiogenic niche that feeds back onto RG, reinforcing progenitor maintenance, modulating differentiation trajectories, and coupling vascular expansion to cortical growth (Vogenstahl et al., 2022). This reciprocal signaling becomes increasingly prominent as endothelial sprouts penetrate progenitor-rich zones and establish sustained physical and molecular interfaces with the VZ and SVZ compartments. ECs regulate the local availability of metabolites and oxygen, and shape the vascular basement membrane, which, in turn, regulates RG behavior. Furthermore, ECs express signaling molecules recognized by RG that directly influence proliferation, fate decisions, and survival (Figure 1).

The role of VegfA signaling in endothelial cell biology is well characterized. However, RG express Vegf receptors 1 and 2 (VegfR1, VegfR2), raising the possibility that EC-derived Vegf could influence NPC populations (Breier et al., 1992; Zhang et al., 2003). *In vitro* experiments have demonstrated neurotrophic and neuroprotective effects of Vegf signaling. Vegf stimulates proliferation in VegfR2-expressing NPCs (Jin et al., 2002) and acts as a guidance cue for migrating NPCs in combination with Fgf2 through a VegfR2-mediated mechanism (Zhang et al., 2003). However, because NPCs also express Vegf, the specific contribution of EC-derived Vegf to these effects remained uncertain.

EC-NPC co-culture experiments began to unravel the cell-type-specific contributions (Shen et al., 2004). These pioneering experiments carried out in the Temple lab in the early 2000s were part of a series of *in vitro* studies that are pivotal to our understanding of intrinsic and extrinsic influences on RG behavior. EC co-culture stimulated embryonic NPC self-renewal, inhibited differentiation, and ultimately increased neuron production. Soluble factors from ECs, including Vegf, were identified as eliciting these effects in combination with direct NPC-EC cell–cell contacts that activated the Notch signaling pathway (Shen et al., 2004).

The role of Notch signaling in RG has been extensively studied, and Notch-mediated lateral inhibition is critical for cortical development (Gonzalez and Reinberg, 2025; Chiba, 2006). However, the contribution of EC-derived Notch ligands is less well studied. Given that endothelial tip cells express Delta-like 4 (Dll4) (Suchting et al., 2007; Hellström et al., 2007) and that EC filopodia directly contact RG (Penna et al., 2021; Wilsch-Bräuninger et al., 2024; Di Marco et al., 2020; Komabayashi-Suzuki et al., 2019), direct interactions between Dll4-expressing filopodia and Notch-expressing RG may contribute to these effects (Figure 1).

### 2.5 Physical interactions with endothelial cells influence radial glial character

As we have alluded to, direct physical interactions between ECs and RG further shape progenitor behavior. Detailed characterizations of blood vessel density in progenitor zones in the mouse cortex suggest RG do not form physical contacts with vessel walls (Komabayashi-Suzuki et al., 2019; Javaherian and Kriegstein, 2009). However, endothelial tip cells protrude filopodia towards the VZ RG in the mouse and human cortex (Figure 1) (Di Marco et al., 2020; Komabayashi-Suzuki et al., 2019). In the embryonic mouse and human ventral telencephalon, EC filopodia have been shown to preferentially contact mitotic RG, and increased filopodia density prolongs mitosis, leading to increased neurogenic divisions (Di Marco et al., 2020). In turn, mitotic RG induce VegfA-mediated filopodia growth (Figure 1) (Di Marco et al., 2020). This study highlights another intricate interaction between EC subtypes and RG and given the close interaction between cortical RG and endothelial filopodia, a similar reciprocal relationship likely exists in the developing cortex.

A 2021 study described protrusions from cortical ECs that preferentially contacted RG; however, these protrusions were reported to arise from vessel walls and were distinct from tip cell filopodia (Penna et al., 2021). A fascinating ultra-structural study published in 2024 revealed that EC protrusions can actually enter, traverse the cytoplasm, and exit RG (Figure 1) (Wilsch-Bräuninger et al., 2024). This study was unable to categorize these protrusions as tip cell filopodia or the EC protrusions seen by Penna et al. (2021); however, the authors postulate that they belong to the former cell type. While the cell-type origin of these protrusions remains unresolved, these studies introduce a new level of crosstalk between ECs and RG. The importance of membrane-membrane contact-mediated signaling and the factors involved remain to be explored.

As cortical development progresses, RG-EC communication appears to transition from predominantly diffusible, long-range signaling to more localized, contact-dependent interactions. This shift likely reflects the transition from signaling to specialization and changing biological needs. Contact-dependent mechanisms provide greater spatial fine-tuning of responsiveness to signaling molecules, support reciprocal feedback, and prevent inappropriate signaling in an increasingly diverse microenvironment.

## 3 Discussion

Cortical development emerges from tightly coordinated interactions between RG and their evolving microenvironment, with RG positioned at the center of this crosstalk. RG actively orchestrate cortical angiogenesis by establishing angiogenic competence, imposing spatial guidance, and stabilizing nascent vascular networks through temporally regulated molecular and contact-dependent mechanisms. In turn, ECs feed back onto RG, shaping progenitor maintenance, differentiation, and niche organization through metabolic, paracrine, and contact-mediated cues. Together, these reciprocal interactions couple vascular expansion to neurogenic demand, ensuring robust cortical growth while preserving progenitor integrity.

Despite growing appreciation of RG-EC crosstalk, key questions remain unresolved. How RG integrate metabolic and signaling inputs from the vasculature with intrinsic lineage programs, whether these cues are interpreted uniformly across RG subtypes, and how EC-derived signals are prioritized relative to RG-derived cues remain open areas of investigation. These questions are particularly relevant in the human cortex, where progenitor populations are more diverse and include expanded basal RG subtypes that are sparse or absent in rodent models.

Dissecting these mechanisms will be essential not only for understanding normal cortical development but also for clarifying how disruptions in the neurovascular niche contribute to neurodevelopmental and neuropsychiatric disorders. As emerging technologies enable increasingly precise interrogation of lineage-specific cell–cell interactions *in vivo* and *in vitro*, the RG-EC interface stands out as a critical and underexplored axis in shaping the developing cerebral cortex.

Emerging human stem cell-derived organoid systems, including vascularized models, offer unprecedented opportunities to interrogate RG-EC interactions within a human developmental context. Integrating organoid-based approaches with spatial transcriptomics, live imaging, and genetic perturbation will be essential for resolving how neurovascular crosstalk is shaped by progenitor complexity and for understanding how its disruption contributes to human neurodevelopmental disease.
